# Confounding factors in profiling of locus-specific human endogenous retrovirus (HERV) transcript signatures in primary T cells using multi-study-derived datasets

**DOI:** 10.1186/s12920-023-01486-y

**Published:** 2023-04-03

**Authors:** Martin V. Hamann, Maisha Adiba, Ulrike C. Lange

**Affiliations:** 1 Leibniz Institute of Virology (LIV), Hamburg, Germany; 2 Institute for Infection Research and Vaccine Development, University Medical Center Hamburg-Eppendorf, Hamburg, Germany

**Keywords:** Human endogenous retrovirus, HERV signature, T cells, Data analysis, Multi-study

## Abstract

**Background:**

Human endogenous retroviruses (HERV) are repetitive sequence elements and a substantial part of the human genome. Their role in development has been well documented and there is now mounting evidence that dysregulated HERV expression also contributes to various human diseases. While research on HERV elements has in the past been hampered by their high sequence similarity, advanced sequencing technology and analytical tools have empowered the field. For the first time, we are now able to undertake locus-specific HERV analysis, deciphering expression patterns, regulatory networks and biological functions of these elements. To do so, we inevitable rely on omics datasets available through the public domain. However, technical parameters inevitably differ, making inter-study analysis challenging. We here address the issue of confounding factors for profiling locus-specific HERV transcriptomes using datasets from multiple sources.

**Methods:**

We collected RNAseq datasets of CD4 and CD8 primary T cells and extracted HERV expression profiles for 3220 elements, resembling most intact, near full-length proviruses. Looking at sequencing parameters and batch effects, we compared HERV signatures across datasets and determined permissive features for HERV expression analysis from multiple-source data.

**Results:**

We could demonstrate that considering sequencing parameters, sequencing-depth is most influential on HERV signature outcome. Sequencing samples deeper broadens the spectrum of expressed HERV elements. Sequencing mode and read length are secondary parameters. Nevertheless, we find that HERV signatures from smaller RNAseq datasets do reliably reveal most abundantly expressed HERV elements. Overall, HERV signatures between samples and studies overlap substantially, indicating a robust HERV transcript signature in CD4 and CD8 T cells. Moreover, we find that measures of batch effect reduction are critical to uncover genic and HERV expression differences between cell types. After doing so, differences in the HERV transcriptome between ontologically closely related CD4 and CD8 T cells became apparent.

**Conclusion:**

In our systematic approach to determine sequencing and analysis parameters for detection of locus-specific HERV expression, we provide evidence that analysis of RNAseq datasets from multiple studies can aid confidence of biological findings. When generating de novo HERV expression datasets we recommend increased sequence depth ( > = 100 mio reads) compared to standard genic transcriptome pipelines. Finally, batch effect reduction measures need to be implemented to allow for differential expression analysis.

**Supplementary Information:**

The online version contains supplementary material available at 10.1186/s12920-023-01486-y.

## Background

Unique gene expression profiles define identity and activity of human cells in both physiological and pathological contexts. They can be determined by genome-wide analysis of cellular transcripts using high throughput next-generation sequencing (NGS), so called transcriptome profiling. Transcriptomic analysis has vastly evolved since its beginnings in the 1990s and has been fundamental in studying and understanding molecular mechanisms of cell physiology and pathology. Standard transcriptomic studies focus on about 20.000 annotated protein-coding and up to 40.000 non-protein-coding genes present in the human genome. These make up around 4% of the human genomic content. However, transcription can occur genome-wide, also in the vast majority of genomic regions not classically defined as genes. Indeed, mounting evidence suggests, that transcripts emerging from these often disregarded regions contribute actively to cell physiology though regulatory or instructive roles [1–4].

Human endogenous retroviruses (HERVs) are evolutionary acquired genomic elements derived from retroviral germline infections [5]. HERVs classify as one type of transposable element and occupy a notable 8–10% of the human genome [6]. They all derive from a proviral structure, that originally consisted of the viral gag, pro, pol and env genes flanked by two long terminal repeats (LTR) containing regulatory elements such as promoter, poly-adenylation signals and multiple binding sites for nuclear proteins. Today, the majority of HERVs exist as fragmented remnants of this structure, often solitary LTRs [5, 7]. Notably, HERV elements show a very high degree of sequence similarity, in particular within HERV families. These families consist of 100s to thousands of single elements with different lengths dispersed throughout the genome. HERVs thus classify as part of the repetitive genome [2, 7–9].

Numerous studies have shown that HERV families are transcribed in human tissues in development, health and disease [10–14]. Depending on the structural arrangement of the HERV element, transcription can generate non-coding as well as protein-coding RNA. In addition, even transcriptional activity arising from solo-LTRs or indeed their active repression can impact on transcript levels of human genes in physical proximity [15–17]. HERVs have thus been associated with various biological processes, e.g. placentation and maintenance of stemness in development, aging and innate immune responses, cancerogenesis, neurodegeneration and autoimmune activity [10, 17–23]. In many cases, these findings have been based on technical assays such as quantitative PCR or RNA expression microarrays that fail to address locus-specific genome-wide transcription patterns. Analysis of the HERV transcriptome at genome-wide level through NGS-based RNA sequencing (RNAseq), has been hampered by the repetitive sequence nature of HERV elements. With no possible clear assignment to a genomic source, ambiguous reads are traditionally disregarded and excluded from transcriptome analysis, making comprehensive HERV transcriptomics unattainable.

To overcome this issue, different bioinformatic tools dedicated to HERV RNAseq data have recently been described that aid with mapping of ambiguous transcript reads [24, 25]. These tools use statistical approaches based for example on the Bayesian mixture model or heuristic approaches, implementing specific filtering criteria for transcript mapping. Mapping is done on specific HERV loci annotations, often manually curated, that specify genomic positions of HERV elements. By implementing these tools, first studies on comprehensive genome-wide locus-specific analyses of HERV transcription have been undertaken [24–26]. They demonstrate a cell-type and disease-specific pattern of HERV transcriptional activity, reminiscent of the unique and state-specific cellular transcriptome of classical genes [25]. These studies also begin to show an intriguing complexity of HERV and host gene interplay. For example, deregulation of HERVs in acute myeloid leukemia appears to alter adjacent gene expression through exposure of HERV-inherent enhancers, promoting oncogenesis [27]. On the other hand, activation of HERV elements in various solid cancer types has been demonstrated to upregulate transcriptional suppressors of the Krüppel-associated box domain-containing zinc-finger protein family (KZFPs) encoded adjacent to deregulated HERVs. This in turn was associated with tumor suppression and improved disease conditions [28]. As for viral infections, locus-specific HERV transcriptome signatures have been proposed to differentiate between cellular infections with distinct viruses, again indicating a complex and locus-specific HERV/host interplay [26].

These data argue that genome-wide HERV transcriptome studies could provide new insights into the complexity of human genome function at a level so far unexplored. HERV transcriptomics could lead to a better understanding of human pathology, aiding with the quest for disease-specific biomarkers and therapeutic targets. In future, studies are hence likely to be focusing increasingly on the contribution of HERV elements to cell physiology. This will require extensive mining of RNAseq datasets. Since RNAseq experiments are costly, require considerable technical skill and source materials can be rare, the community will rely heavily on the wide array of datasets already available in the public domain. However, RNAseq datasets are not per se standardized as to technical parameters and quality of input material. They differ in depth of coverage, i.e. the number of reads per sample collected within one sequencing run, and in read lengths, i.e. the number of base pairs (bp) read at a time. Furthermore single-end versus paired-end reading can be distinguished, specifying whether sequencing is done from one or both ends of the cDNA fragment. Standard RNAseq experiments vary between 20 million (mio) up to 200 mio read depth with 50 to 150 bp read length, using single or paired end technology. Quality of the input material also differs greatly and is generally assessed using established quality control parameters (e.g., phred score per bp, PCR duplicated reads, read length distribution, percentage of mappable reads). For analysis of cellular genes, certain optimal technical parameters have been empirically determined depending on the query. For HERV transcriptomic analysis however, it is largely unknown how technical specifics of the RNAseq dataset impact on the results. While there are indications that for example single and paired-end technologies might influence outcomes [24, 29], a detailed, comprehensive analysis in this context is lacking.

To address this issue, we have undertaken HERV transcriptome analysis of primary CD4 and CD8 T cells, using several publicly available RNAseq datasets with differing technical parameters. Our analysis is based on the ERVmap tool, determining expression of 3220 near full-length HERV elements from 3 different HERV classes (12 supergroups, 71 groups) [7, 25]. We focus in particular on how differences in sequencing depth and read length impact on the recovered HERV transcriptome and whether datasets of the same cell type lead to comparable results between different studies.

## Methods

### RNA-seq datasets

We obtained RNA sequencing datasets from multiple studies via the NCBI Sequence Read Archive (SRA) using the SRA Toolkit v3.00 (https://trace.ncbi.nlm.nih.gov/Traces/sra/sra.cgi?view=software SRA Toolkit Development Team). Dataset descriptions are provided in Table 1. Dataset accession numbers: Tan et al. [30] (SRR11031268, SRR11031269, SRR11031270, SRR11031271, SRR11031272, SRR11031273, SRR11031274, SRR11031275, SRR11031276); DFG (SRR12095608, SRR12095609, SRR12095616, SRR12095617); Lopusna et al. [31] (SRR12224910.16 (combined SRR12224910 to SRR12224916), SRR12224917, SRR12224918.24 (combined SRR12224918 to SRR12224924), SRR12224925); Linsley et al. [32] (SRR1550989, SRR1550990, SRR1551050, SRR1551051, SRR1551057, SRR1551058, SRR1551071, SRR1551072); White et al. [33] (SRR5891091, SRR5891092, SRR5891093, SRR5891094); UWashington.HREMP (SRR643766, SRR644512, SRR644513, SRR644514, SRR453391, SRR980471); Bediaga et al. [34] (SRR8534322, SRR8534326, SRR8534327, SRR8534328); ENCODE.SUNY-Albany (SRR3192487, SRR3192488, SRR3192489); CSHL (SRR307911.2 (combined SRR307911 and SRR307912)); Caltech(SRR521477.84 (combined SRR521477 to SRR521484), SRR521501.2 (combined SRR521501 and SRR521502), SRR52150, SRR521513.5 (combined SRR521513 to SRR521515)).

**Table 1:** Summary of RNAseq dataset parameters for each study

Study	year of data deposition	link to bioproject	platform	instrument	seqmode (bp)	cell type (no. donor x replicates)	matched donor samples	input reads (mean ± SD)	filtered reads (mean ± SD)	% HERV reads of filtered reads (mean ± SD)	no exp. HERVs (mean ± SD)	note	donor_number
***Tan et al.***	2020	https://www.ncbi.nlm.nih.gov/bioproject/PRJNA605014	ILLUMINA	NextSeq500	1 × 75	CD4 (3 × 3)	no	3.81E + 07 ± 2.19E + 07	2.11E + 07 ± 1.26E + 07	0.21 ± 0.02	597 ± 100.78	water	
***DFG***	2020	https://www.ncbi.nlm.nih.gov/bioproject/PRJNA642003	ILLUMINA	Hiseq2500	1 × 50	CD4 (2 × 1) / CD8 (2 × 1)	yes	2.80E + 07 ± 2.01E + 07	1.71E + 07 ± 1.05E + 07	0.25 ± 0.01	811.75 ± 33.36	umbilical cord blood	Donor_9/10
***Lopusna et al.***	2021	https://www.ncbi.nlm.nih.gov/bioproject/PRJNA646366	ILLUMINA	NovaSeq 6000	2 × 150	CD4 (2 × 1) / CD8 (2 × 1)	no	1.35E + 08 ± 8.13E + 07	5.84E + 07 ± 3.08E + 07	0.31 ± 0.01	1033.25 ± 127.70		
***Linsley et al.***	2014	https://www.ncbi.nlm.nih.gov/bioproject/PRJNA258216	ILLUMINA	HiScanSQ	2 × 50	CD4 (4 × 1) / CD8 (4 × 1)	yes	3.80E + 07 ± 6.87E + 06	1.82E + 07 ± 2.53E + 06	0.22 ± 0.01	500.63 ± 28.35		Donor_5/6/7/8
***White et al.***	2018	https://www.ncbi.nlm.nih.gov/bioproject/PRJNA396949	ILLUMINA	HiSeq 2000	2 × 50	CD4 (4 × 1)	no	2.06E + 08 ± 1.22E + 07	9.72E + 07 ± 7.20E + 06	0.23 ± 0.01	1094.75 ± 96.13	DMSO	
***University of Washington Human Reference Epigenome Mapping Project (UWashington.HREMP)***	2013	https://www.ncbi.nlm.nih.gov/gds/200018927	ILLUMINA	HiSeq 2000	2 × 75	CD4 (2 × 1) / CD8 (2 × 1)	yes	3.96E + 08 ± 1.20E + 08	2.15E + 08 ± 5.49E + 07	0.31 ± 0.02	1705.75 ± 440.47		Donor_1/2
***Bediaga et al.***	2021	https://www.ncbi.nlm.nih.gov/bioproject/PRJNA521046	ILLUMINA	NextSeq500	2 × 80	CD4 (2 × 1) / CD8 (2 × 1)	yes	1.36E + 08 ± 7.00E + 07	8.27E + 07 ± 4.21E + 07	0.28 ± 0.01	1013.25 ± 147.22		Donor_3/4
***University of Washington Human Reference Epigenome Mapping Project (UWashington.HREMP)***	2012	https://www.ncbi.nlm.nih.gov/gds/200018927	ILLUMINA	HiSeq 2000	2 × 75	CD34 (1 × 1)	no	5.35E + 08	2.42E + 08	0.35	1340	cord blood	
***University of Washington Human Reference Epigenome Mapping Project (UWashington.HREMP)***	2013	https://www.ncbi.nlm.nih.gov/gds/200018927	ILLUMINA	HiSeq 2000	2 × 75	CD19 (1 × 1)	yes	5.36E + 08	2.87E + 08	0.27	1494		Donor_2
***ENCODE/SUNY Albany***	2016	https://www.ncbi.nlm.nih.gov/bioproject/PRJNA30709	ILLUMINA	Genome Analyzer IIx	2 × 75	Keratinocytes (1 × 3)	no	2.84E + 08 ± 2.88E + 06	1.44E + 08 ± 4.16E + 06	0.17 ± 0.02	1085.67 ± 135.24		
***CSHL***	2011	https://www.ncbi.nlm.nih.gov/geo/query/acc.cgi?acc=GSM758566	ILLUMINA	Genome Analyzer IIx	2 × 75	H1-hESC (1 × 1)	no	1.62E + 08	8.37E + 07	1.82	1786		
***Caltech***	2012	https://trace.ncbi.nlm.nih.gov/Traces/?view=study&acc=SRP014320	ILLUMINA	Genome Analyzer	1 × (1x75), 2 × (2x75)	H1-hESC (3 × 1)	no	1.91E + 08 ± 1.45E + 08	7.01E + 07 ± 6.22E + 07	1.94 ± 0.52	1543.33 ± 349.81		

### Dataset Quality Control

Datasets derived from one biological sample available as multiple files in the SRA database were combined using the Unix ‘cat’ function, prior to read mapping. Initial dataset quality was visualized using FastQC and MultiQC reports [35, 36]. Subsequently, Illumina reads were quality trimmed using TrimGalore! (v0.6.4; https://github.com/FelixKrueger/TrimGalore), removing low quality reads and sequencing adapter in automatic detection mode. These quality validated fastq files went into downstream read alignment pipelines.

### HERV expression quantification using ERVmap pipeline

Read mapping was done on the human genome reference build GRCh38 (hg38). HERV expression analysis was performed on the 3220 near-full length HERV elements, gathered in Tokuyama et al. [25], since the chance of detecting HERV transcripts is highest in these elements compared to solo-LTR elements for instance.

Reads were aligned with Burrows-Wheeler Aligner (BWA v0.7.17) using standard settings (‘bwa mem’) [37]. Subsequently, mapped reads with high accuracy where filtered following the ERVmap criteria and using the original, unmodified ERVmap perl script parsing the CIGAR field of mapped reads [25] (https://github.com/mtokuyama/ERVmap). In summary, the script filters for reads that have (i) one best match for alignment, (ii) the second best match must have at least one additional mismatch and (iii) must not have more than X mismatches in total (X is calculated relative to read length of sequence data; i.e. X equals 3 in 150 bp paired-end reads)[25]. Next, SAM to BAM file conversion and processing was performed using samtools (v1.10) ’view’, ’sort’ and ’index’ commands [38]. With bedtools (v2.5.1) [39] function ’coverage’ raw read counts for the 3320 near full-length HERV elements were obtained. All datasets were analyzed using this ERVmap pipeline, whereas selected datasets were additionally analyzed using the Telescope pipeline (Figure S3).

### HERV expression quantification using Telescope pipeline

Another pipeline that was used for HERV expression analysis was the Telescope pipeline, which contains a reference annotation containing 14,968 manually curated HERV loci and is designed for solving multimapping reads [24]. These HERV loci are defined by combining RepeatMasker annotations located in adjacent or nearby genomic regions, and belonging to the same HERV subfamily (https://github.com/mlbendall/telescope_annotation_db)(24). It uses a generative model of RNA-seq for reassigning the ambiguously mapped fragments to the most probable source transcript, and thus addresses the uncertainty in fragment assignment [24]. Here, first reads were subjected to a very sensitive local alignment (--very-sensitive-local) to the human reference genome hg38 using Bowtie 2 with a minimum alignment score threshold of 95% (--score-min L,0,1.6) along with a maximum of 100 alignments per reads (-k 100)[24, 40]. The mapped BAM files were then analyzed using Telescope, which includes Bayesian reassignment and up to 200 iterations of the expectation-maximization algorithm[24]. Finally, from the resulting report, “final counts” columns were retrieved, which represented the HERV count data.

### Gene expression quantification

Read mapping was done on the human genome reference build GRCh38 (hg38). Reads were mapped with HISAT2 (v2.1.0) [41]. SAM to BAM file conversion was handled as stated above using samtools (v1.10) ’view’, ’sort’ and ’index’ commands [38]. Finally, raw cellular transcript counts were quantified using the HTSeq-count tool (v0.13.5) [42].

### Expression data analysis

Further downstream analysis and visualization was performed in R (v4.2.0) including the packages DESeq2 (v1.36.0) [43], limma (v3.52.4) [44], ggplot2 (v3.3.6), pheatmap (v1.0.12), ggVennDiagram (v1.2.0) and plot_matrix (v1.6.2). DESeq2 read normalization method (median of ratios) was used on cellular gene transcript counts to obtain size factors for each dataset. These size factors were then applied to HERV transcript counts, allowing comparison between samples [25, 43, 45].

## Results

### Genome-wide detection of HERV transcripts in primary human T cells is susceptible to technical parameters of RNAseq datasets

The primary goal of our study was to understand how different RNAseq datasets perform for HERV transcriptomic analysis. We decided to focus on two well-characterized human immune cell types, namely CD4 + and CD8 + T cells. For both cell types, a considerable number of RNAseq datasets is available in the public domain. In addition, we selected primary cell types for increased translatability. We mined public repositories and retrieved 25 RNAseq datasets from 7 studies for primary, non-activated CD4 + T cells and 12 datasets from 5 studies for primary, non-activated CD8 + T cells (study details summarized in Table 1). Our dataset collection includes samples from larger sequencing consortia (Human Reference Epigenome Mapping Project (HREMP) & ENCODE) as well as datasets from smaller research groups. All studies applied Illumina-based sequencing technology but utilizing different platforms and technical parameters (Table 1). All but two studies used paired-end sequencing (Table 1). All but two studies (T cell isolation from cord blood) had lymphocytes isolated from peripheral blood samples. In five studies, CD4 and CD8 T cells were isolated from the same donor (matching datasets). Depth of sequencing ranged between 18,7 and 546,5 mio reads per sample (mean ± SD: 115,0 ± 128,3 mio) and read lengths ranged from 50 to 150 bp per reads.

Datasets were subsequently analyzed to retrieve HERV transcripts using ERVmap as previously described [25]. This pipeline allows for genome-wide locus-specific HERV expression analysis based on stringent filtering criteria for mapping. In essence, reads must be uniquely mapped to the reference genome with high confidence and the second-best match must have at least one additional mismatch in sequence alignment. A manually curated annotation of 3220 near-full length HERVs (average 7,5 kb in length) was used [25]. In parallel, each dataset was analyzed for cellular gene transcripts using standard sequence read alignment and quantification tools.

**Fig. 1:**
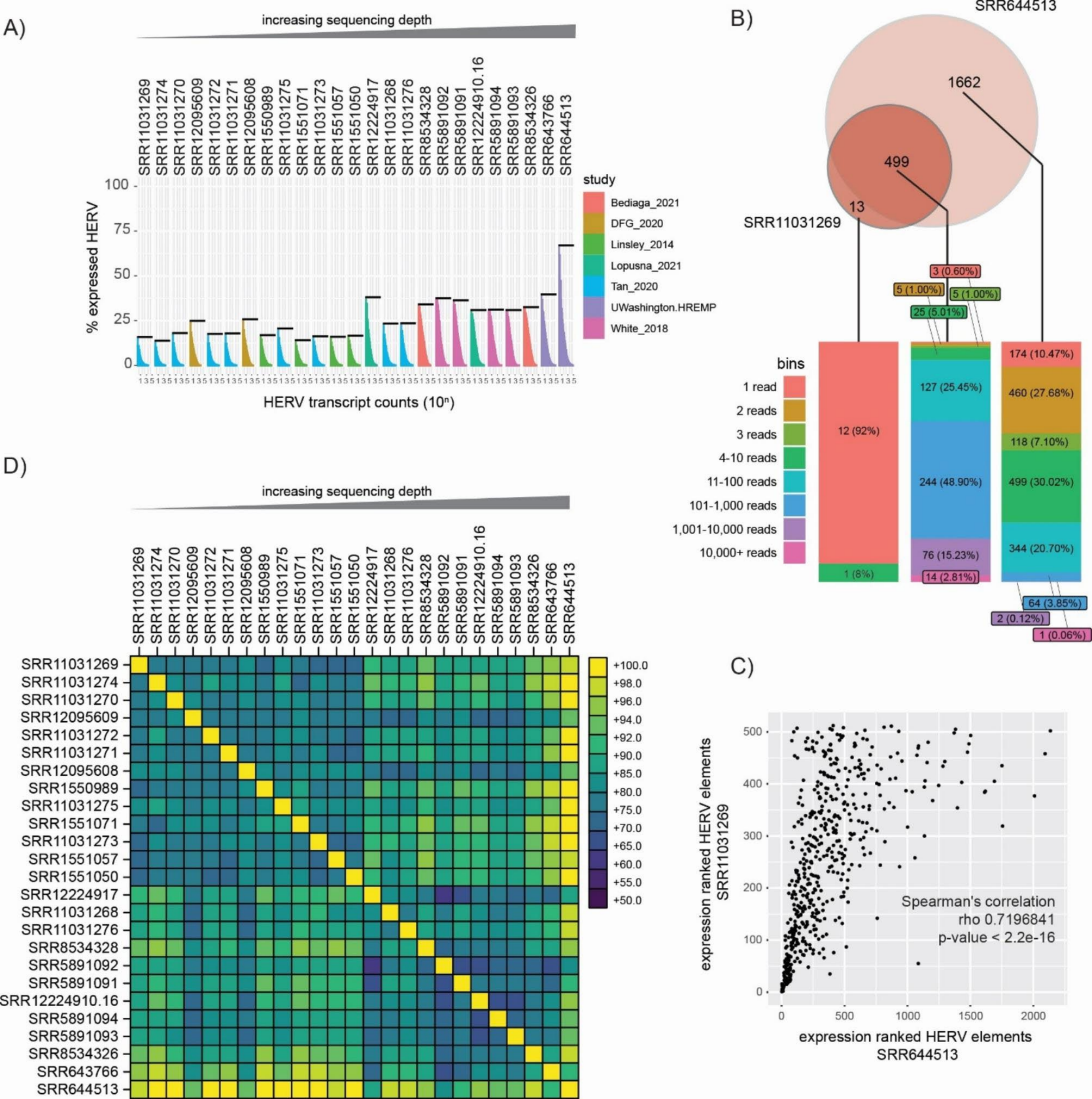
HERV expression in primary CD4 + T cells. **(A)** Raw HERV transcript counts are plotted for each HERV element. A HERV element is considered to be expressed with at least one read being mapped to the HERV loci. Black line indicates % of expressed HERVs per dataset. Datasets are identified by SRA database numbers and ordered by increasing sequencing depth. **(B)** Qualitative comparison of expressed HERV elements between datasets with least and highest sequencing depth. Absolute number of expressed HERV elements are presented in the Venn diagram. Bar chart below depicts distribution of mapped reads per HERV element for each Venn section. **(C)** Ranked HERV expression comparison between datasets from B. The Spearman correlation coefficient and p-value is indicated. **(D)** Pairwise comparison matrix presenting the overlap of expressed HERV elements between datasets. Order of datasets equivalent to panel A

We first addressed the question of how different RNAseq datasets with differing technical parameters perform in quantitative detection of locus-specific HERV expression. In particular, we asked how RNAseq datasets with low sequencing depth, i.e. 20 mio reads per sample as recognized standard for cellular transcriptomics analysis, could deliver. We found that expression of HERVs could be detected in all datasets, ranging between 13,8% and 67,1% of annotated HERV loci (mean ± SD: 26,9% ± 12,7%) (Fig. 1A; Figure S1A). This finding is in line with previous data, that show around 50% overall HERV expression levels in different primary cells [25]. As expected, datasets with higher sequencing depth showed higher relative number of expressed HERV as compared to datasets with lower sequencing depth (Fig. 1A & S1A). We did not find differences between CD4 + and CD8 + T cells concerning quantitative HERV expression (Figure S1E). Hence, locus-specific HERV expression can be detected also in datasets with low sequencing depth.

We next asked, to which extent HERV transcriptomes derived from low sequencing depth datasets could reflect HERV expression signatures derived from deep-sequenced sets. We defined a HERV element as being expressed, if at least one read was mapped in the ERVmap pipeline. Next, we qualitatively compared the set of expressed HERVs in the dataset with the lowest sequencing depth (18,7 mio reads (CD4+, SRR11031269) / 25,8 mio reads (CD8+, SRR12095616)) to the dataset with highest sequencing depth (481 mio reads (CD4+, SRR644513) / 547 mio reads (CD8+, SRR644514)) (Fig. 1B, Figure S1B). For CD4 + T cells, we observed that transcripts for 13 (2,5%) HERVs were solely detected in the smaller dataset, while transcripts for 499 (97,5%) HERVs were detected in both datasets. Transcripts for additional 1662 HERV loci were only detected in the larger dataset. For CD8+, 67 (7,9%) HERVs were solely detected in the smaller dataset, while transcripts for 785 (92,1%) HERVs were detected in both datasets. Additional transcripts for 1346 HERV loci were only detected in the larger dataset. This suggests that HERV expression derived from datasets with low sequencing depth can reflect the majority of expressed HERVs as detected in datasets with more than 20-fold greater sequencing depth. To assess whether the overlap in expressed HERV elements correlates with expression levels, we ranked HERV elements from most to least expressed based on associated read counts (Fig. 1C and S1C). Statistical analysis using Spearman’s coefficient reveals positive correlation between HERV expression levels in both datasets (Spearman’s coefficient CD4 + 0,719; CD8 + 0,697), indicating that indeed RNAseq datasets with low sequencing depth allow faithful detection of most abundantly expressed HERV elements.

Comparison of the dataset with the lowest sequencing depth to the dataset with highest sequencing depth also revealed a subset of HERV transcripts solely detected in the low or high depth dataset (Fig. 1B and S1B). Whereas HERV elements detected in the high depth dataset only could be explained by greater transcript depth, the finding that low depth datasets show uniquely transcribed HERV elements was somewhat not anticipated. To explore this further, we stratified the data according to the number of mapped reads for these elements. This analysis revealed that most transcripts detected in the low depth dataset just met the threshold level of one mapped read (12 out of 13 (92%) in CD4 + T, 46 out of 67 (68,7%) in CD8 + T cells). Considering the low chosen threshold for expression (> 0 mapped reads), it is hence plausible that these HERVs were detected as artefacts and are not actually expresssed. Most HERV elements detected solely in the high depth datasets showed higher read counts supporting their status as transcribed elements (Fig. 1B and S1B).

To circumvent this potential drawback, we next set the threshold level of expression to > 1 or > 2 mapped reads in our analysis. This resulted as expected in an overall decrease of detected HERV elements, which was however strongest in the intersect of unique low depth dataset-expressed HERVs (Figure S3). Nevertheless, a small fraction of HERV elements solely detected in the low depth datasets demonstrated a substantial number of reads (one element with 5 mapped reads in CD4 T cells; 10 elements with 3 to 19 mapped reads in CD8 T cells). We therefore would consider these elements to be actually expressed in the dataset, reasoning that lack of their detection in high depth datasets is likely a result of dataset-inherent differences due to for example sample handling prior and during sequencing.

To verify that potential artefacts of HERV expression in low depth datasets are not specific to applied ERVmap analysis pipeline, we next re-analysed low and high-depth sequenced datasets using the Telescope pipeline [24]. In contrast to ERVmap, Telescope was developed to map ambiguous reads utilizing a statistical expectation-maximization algorithm and also contains a broader annotation list comprising 14,968 individual HERV elements. In agreement with our results obtained using ERVmap, we found that Telescope also calls a small number of HERV elements that are solely expressed in low sequencing depth datasets for different expression thresholds (Figure S3). This observation argues against an analysis pipeline-specific effect.

To further compare HERV expression profiles among all datasets, we generated a pairwise comparison matrix (Fig. 1D; Figure S1D). We observed an overlap of at least 64% and up to 99% among expressed HERVs. As indicated in our previous finding, datasets with low sequencing depth and therefore relatively low number of expressed HERVs showed highest percent overlap with datasets sequenced deepest.

Taken together, our data show that while the extent of HERV transcript detection in RNAseq datasets increases with sequencing depth, datasets with low sequencing depth, such as standard cellular transcriptome analysis can still be used for detection of most prominently expressed HERV elements. In general, we made similar observations for HERV transcriptome analysis in CD4 + T cell and CD8 + T cell RNAseq datasets, arguing that our findings are not cell type-specific (Figure S1).

### Sequencing depth over read length and seqmode as decisive RNAseq parameter for HERV transcriptomics

**Fig. 2:**
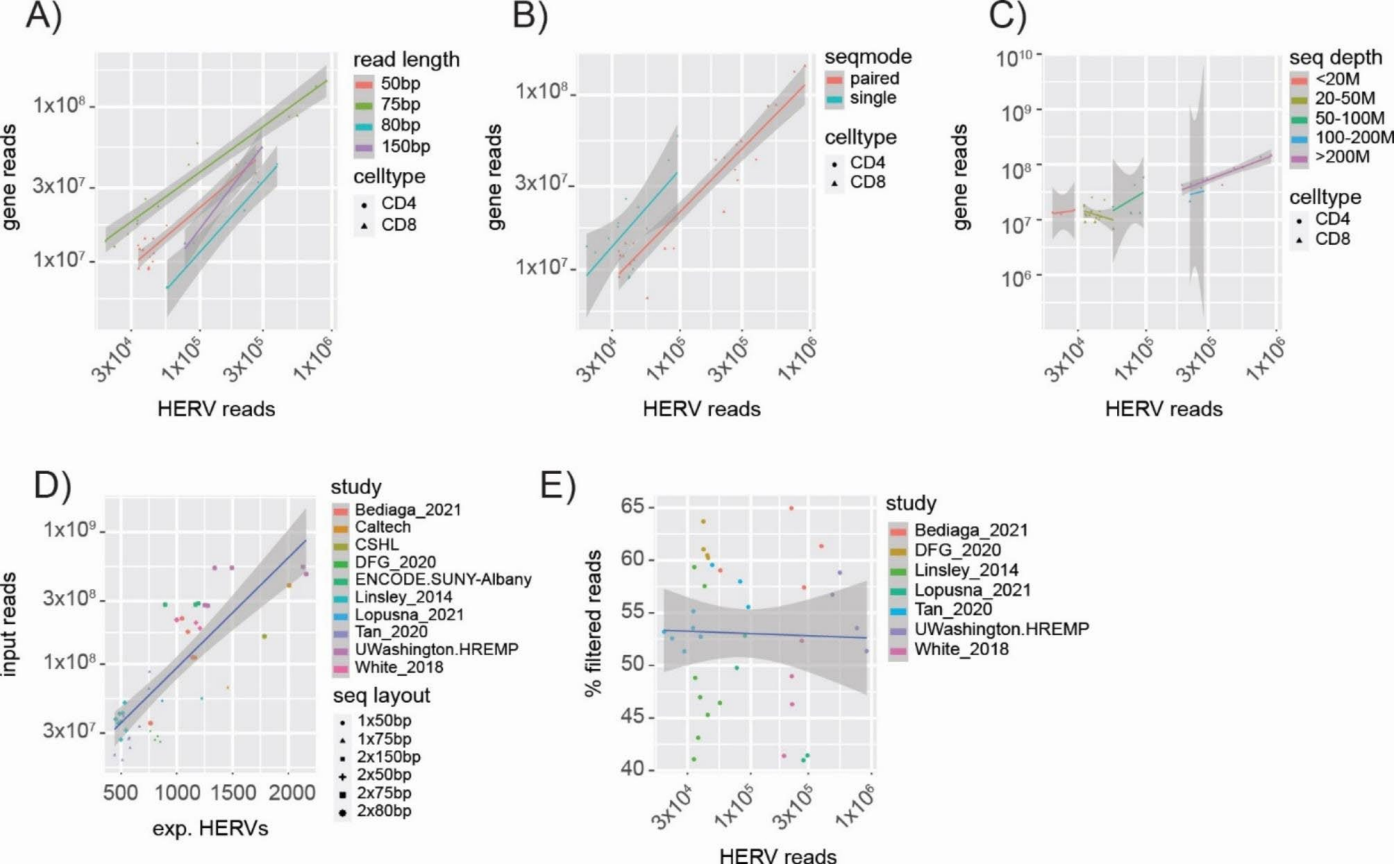
Sequencing parameter impact on HERV transcriptome mapping in primary CD4 + and CD8 + T cells. Raw mapped HERV and gene transcript counts are plotted and grouped by sequencing parameter read length **(A)**, seqmode **(B)** and sequencing depth **(C)**.** (D)** Correlation plot between sequencing depth (input read number) and the number of expressed HERV elements. A HERV element is considered to be expressed with at least one read being mapped to the HERV loci. **(E)** Correlation of dataset quality (i.e. the fraction of high quality mapped reads) versus the raw count of mapped HERV reads

We next asked, how individual technical parameters of RNAseq datasets might impact on HERV transcript detection and compared our findings to detection of cellular gene transcripts. We plotted the number of raw HERV reads versus cellular gene reads for each dataset, whereas datasets were grouped by sequencing parameters, i.e. read length, seqmode (single- vs. paired-end sequencing) and sequencing depth (Fig. 2 and S2). Each study contributed multiple datasets of the same technical parameters.

As expected, for all technical parameter groupings, we observed a positive correlation between mapped HERV and cellular transcripts in all datasets (Fig. 2). Both read length and seqmode had little influence on the level of detected HERV transcripts in relation to cellular transcripts (Fig. 2A and B). However, increasing sequencing depth clearly associated with an increase in mapped HERV transcripts as well as cellular transcripts (Fig. 2C). This correlates well with the increased number of expressed HERVs in datasets with high numbers of input reads (Fig. 2D). Taken together, sequencing depth of RNAseq datasets appears to be the critical parameter that positively impacts on the number of detectable locus-specific HERV transcripts.

We furthermore examined, how different datasets performed for quality and if this affected HERV transcript detection. The percentage of high-quality mapped reads (% filtered reads) that were used for HERV transcript analysis was used as quality parameter for each dataset. This parameter reflects for example quality deviations derived from RNA extraction, cDNA synthesis and library preparation. We found that 40–60% of input reads were mapped with high confidence in all datasets across all studies (Fig. 2E), which indicates an overall similar quality of data. Within this range, no correlation of quality score with number of detectable locus-specific HERV reads was observed (Fig. 2E). This finding indicates that dataset quality was not a confounding parameter in our multi-study analysis.

We also extended our analysis to a limited number of RNAseq datasets derived from additional cell types namely CD19 + and CD34 + immune cells, keratinocytes and human embryonic stem cells (H1). We obtained comparable results to our data on CD4 + and CD8 + T cells, indicating that our findings are independent of cell type (Figure S2). In summary, for comprehensive loci-specific HERV transcriptomics, our analyses indicate that a sequencing depth of or above 100 mio reads per sample is most likely to yield best results (Fig. 2C), while read length and seqmode are secondary.

### Study-dependent confounding factors necessitate batch effect reduction for analysis of HERV transcriptome signatures

**Fig. 3:**
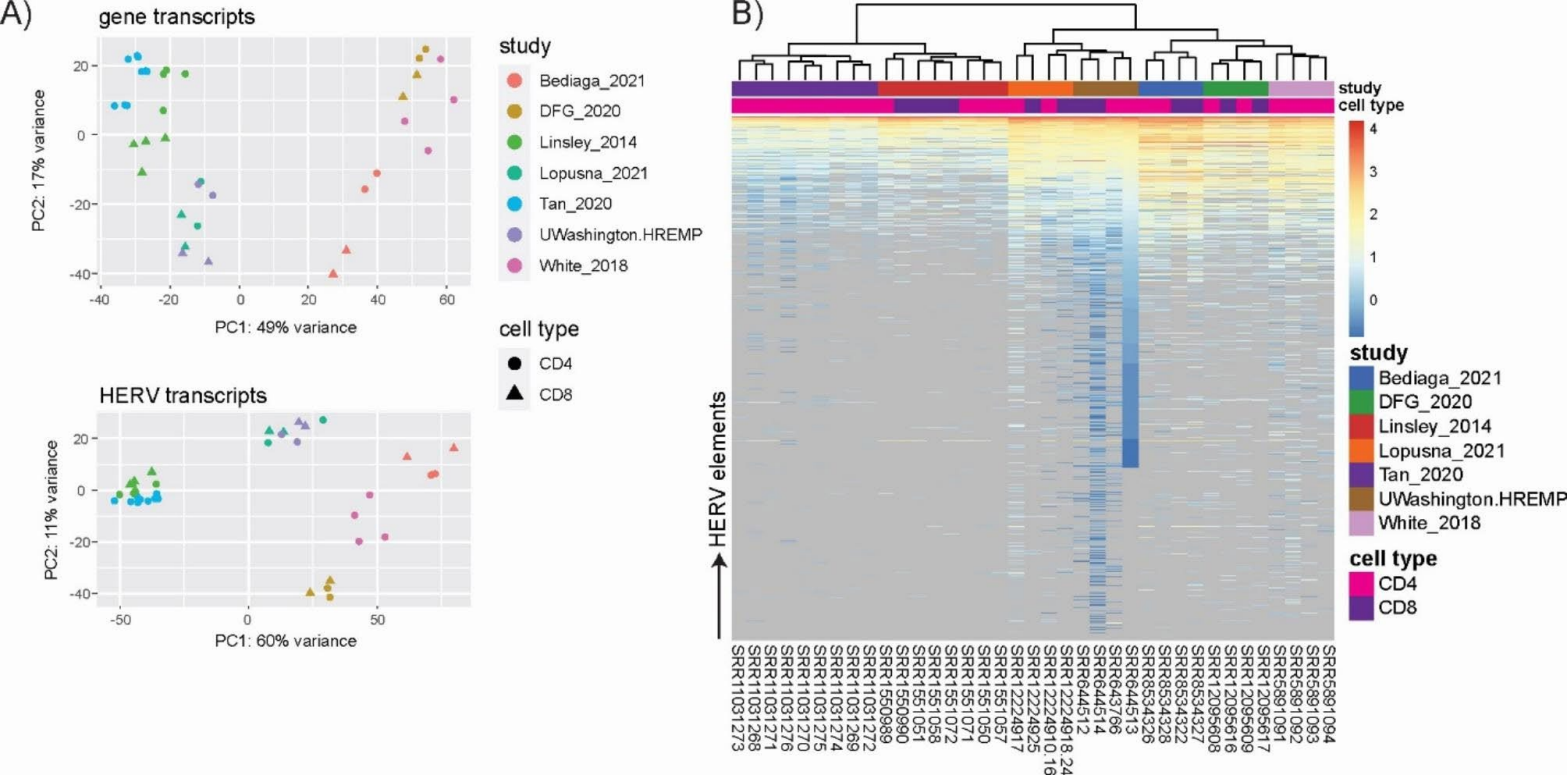
Normalized read counts of CD4 + and CD8 + T cell derived HERV and gene transcripts without batch correction. **(A)** Principal component analysis based on HERV and gene transcripts. **(B)** Hierarchical cluster analysis and heatmap of HERV transcripts. Counts are log10 transformed, zero counts are depicted in grey and count matrix was sorted for deep sequenced dataset SRR644513 (CD4 + T cells, UWashington.HREMP) to increase clarity

We next went on to investigate, if and to which extent HERV transcriptome profiles of the same cell type are comparable when derived from different RNAseq datasets, that reflect technical differences in sequencing parameters and variable study set-ups. This aspect is of particular concern for HERV transcriptome analyses of specific conditions or rare sample types that most often rely on pooling RNAseq datasets from diverse studies. Read counts were normalized with DESeq2 to correct for different sequencing depths. Subsequently, we undertook principle component analysis (PCA) for HERV transcripts and cellular gene transcripts derived from all CD4 + and CD8 + T cell RNAseq datasets. For both transcript types, we saw an obvious clustering of samples according to study origin and not according to cell type origin (Fig. 3A). This result was also observed, when plotting HERV transcriptomes using hierarchical clustering and expression heatmaps: datasets derived from the same study clustered closer than datasets derived from the same cell type (Fig. 3B).

**Fig. 4:**
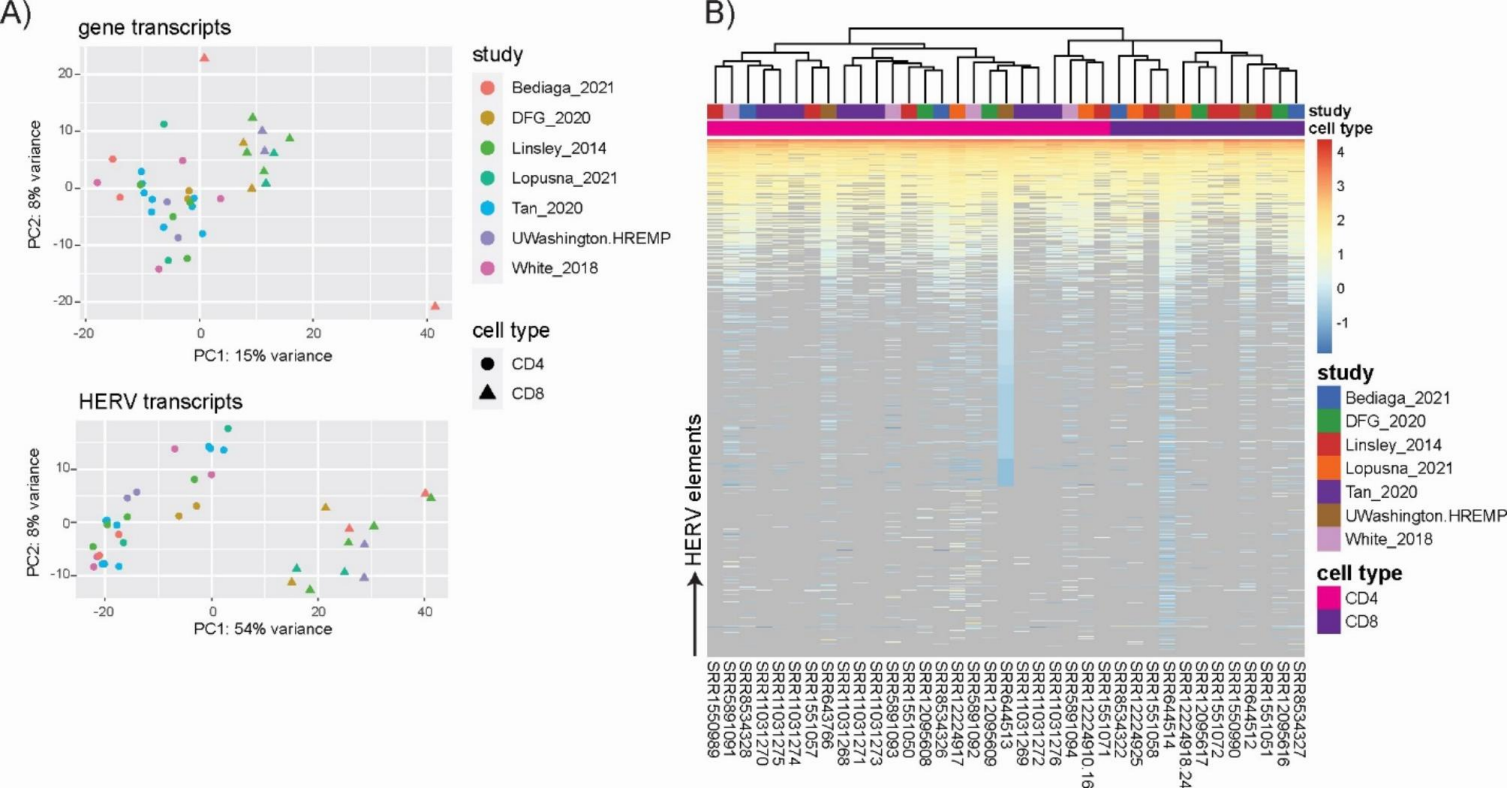
Normalized read counts of CD4 + and CD8 + T cell derived HERV and gene transcripts after batch correcting for inter-study differences. **(A)** Principal component analysis based on HERV and gene transcripts. **(B)** Hierarchical cluster analysis and heatmap of HERV transcripts. Counts are log10 transformed, zero counts are depicted in grey and count matrix was sorted for deep sequenced dataset SRR644513 (CD4 + T cells, UWashington.HREMP) to increase clarity

The phenomenon of batch effect has been well described to confound biological analyses, although scientific publications often remain elusive in this regard [46]. We here show that for HERV transcriptomics batch effects are equally relevant when pooling datasets from multiple studies. To outweigh dataset disparities rooted in inter-study differences, such as for example differences in sample preparation and sequencing conditions, we corrected CD4 and CD8 counts with the limma package function ‘removeBatchEffect()’. These batch-corrected (bc) datasets, were then used for PCA and hierarchical cluster analysis. Figure 4 A demonstrates that for both cellular and HERV transcripts, clustering of bc-samples was now observed in a CD4 + and CD8 + cell-specific manner. Furthermore, hierarchical cluster analysis and expression heatmaps using bc-datasets showed close association according to cell type and not study origin as observed before batch correction (Fig. 4B). Thus, batch effects do affect HERV transcriptomics and HERV transcriptomics studies relying on pooled datasets should be aware of this by including appropriate correction steps.

Noteworthy, we observed that sample clustering in the HERV PCA reflects similar patterns compared to the gene PCA (Figs. 3A and 4 A). This indicates that HERV expression data based on an annotation of 3220 near-full length elements is sufficiently powerful to replicate dataset differences derived from genic transcriptome analysis based on > 55,000 transcripts. In accordance with previous publications [4, 14, 25, 47], this finding strongly supports the hypothesis that cellular identity is not only reflected by a cell-specific transcriptome but also a cell type-specific HERV transcript signature.

### Analysis of inter-donor variability in context of HERV transcriptome signatures

**Fig. 5:**
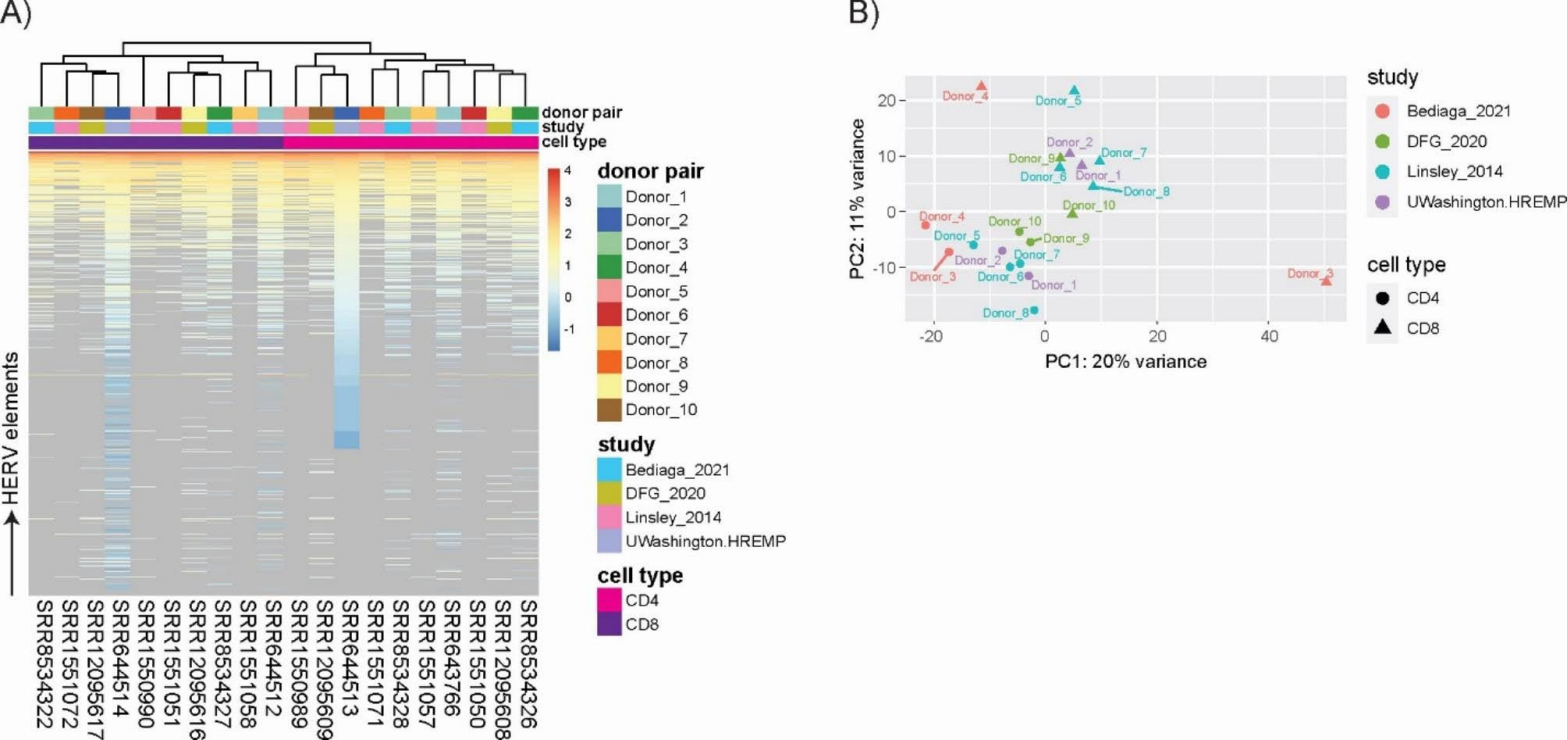
Donor-matched CD4 + and CD8 + T cell datasets and HERV expression. **(A)** Hierarchical cluster analysis and heatmap of HERV transcripts. Counts are log10 transformed, zero counts are depicted in grey and count matrix was sorted for deep sequenced dataset SRR644513 (CD4 + T cells, UWashington.HREMP) to increase clarity. **(B)** Principal component analysis based on HERV transcripts

Since analysis of primary samples often relies on pooling datasets from different biological donors, we next asked to which extent donor variability might impact on detection of cell type-specific HERV transcriptome signatures, especially given the close ontological relation between CD4 + and CD8 + T cells. We obtained 10 donor-matched RNAseq datasets for CD4 + and CD8 + T cells from four of the seven studies included in our analysis. These were submitted to locus-specific HERV transcript detection including batch correction. For all donor pairs, PCA shows clustering of samples according to cell type origin independent of which study the dataset was extracted from (Fig. 5A). The same observation was made, when plotting HERV signatures for donor pairs in hierarchical cluster analysis, where study- and donor features were secondary to cell type in determining signature clusters (Fig. 5B). In summary, inter-donor variability in our datasets is smaller compared to differences in cell-type specific HERV transcriptome profiles. Robust HERV transcriptome profiles are distinguishable for these ontologically closely related T cell types.

## Discussion

The main goal of our study was to clarify, how different RNAseq datasets could be combinational explored to derive locus-specific HERV transcriptome signatures. With increasing evidence that HERV-derived transcripts can impact in different ways on cell physiology, there is rapidly expanding interest in exploring the role of HERV elements both in health and multiple disease conditions, such as cancer, neurological and immunological pathologies [14, 48–56]. HERV transcriptomics will likely evolve as one aspect of disease diagnostics and could potentially serve as biomarker or offer targets for therapeutic approaches. The fast-rising number of publicly available RNAseq datasets supports this development and facilitates HERV research.

We therefore set out to clarify different aspects that need to be taken into consideration when embarking on HERV transcriptomic analysis. We first focused on how technical dataset parameters impact on locus-specific detection of HERV transcripts and found sample sequencing depth to be a critical factor. Our data suggests that > = 100 mio sequencing reads per sample support comprehensive HERV transcriptome analysis. Nevertheless, we also show that datasets with low sequencing depths can be used for detection of most abundant HERVs.

In our study we counted HERV reads, which were mapped uniquely and with high confidence using ERVmap [25]. Thus, a HERV element with one aligned read was regarded expressed. Other established pipelines such as Telescope apply statistical models, i.e. utilizing a Bayesian expectation-maximization algorithm [24] to aid read assignment to highly similar HERV sequences. These methods benefit from making analytical use of more sequencing reads, compared to our conservative approach. We have employed Telescope on a subset of datasets included in this study and found the results comparable to ERVmap. In future, a more comprehensive comparison between single-locus HERV transcriptome pipelines would be helpful to delineate assay-specific strengths and drawbacks and in general improve the quality of future undertakings that aim at detecting HERV expression signatures.

Our analysis revealed a number of expressed HERV elements in low sequencing depth datasets that are not detected in corresponding high sequencing depth datasets. Most of these elements are called by single mapped reads and thus could potentially represent false positives due to the applied low threshold level of one mapped read. This phenomenon is replicated in another analysis pipeline. It can be adjusted by changing the threshold of read counts upon which a HERV element is classified as expressed. However, even after adjustments, a small number of HERV elements supported by a considerable amount of mapped reads, remain to be called expressed only in low depth datasets. It might be questionable to flag these as false positives. Rather, we suggest these to be dataset-inherent differences in HERV expression. These could for example be explained by different procedures of T cell isolation and cultivation as well as RNA sample and sequencing library preparation.

In addition, we found batch effect reduction to be an important step when qualitative analysis is based on datasets from multiple sources. Certainly, batch correction has the potential to mask biological heterogeneity, skewing differential expression analysis [46, 57, 58]. However, for both cellular as well as HERV-derived transcripts, batch effect reduction was necessary to remove confounding parameters originating from technical dataset differences. We used the broadly utilized ‘removeBatchEffect()’ function within the limma R package [44], which resolved prominent sample clustering according to study towards a clear distinction of cell types. This is a prerequisite to downstream differential gene/HERV expression analysis and thus should be included in future studies. Methods to detect and reduce batch effects are under constant improvement, as the field of multi-omics studies moves forward [59–61]. HERV transcriptome studies will very likely benefit from these developments.

In our analysis we focused mainly on > 3200 autonomous HERV sequences, i.e. near full-length HERV sequences predicted to be capable of transcriptional and translational activity [7, 25]. While regulation of this subset of HERVs could arguably be most influential on cellular physiology, it should be noted, that it disregards shorter retroviral mosaic forms and soloLTRs [7]. There are examples that especially soloLTRs can impact on cellular gene regulation [62]. While we have also employed a broader annotation of around 14,000 HERV sequences on a restricted subset of samples in presented study, it remains to be thoroughly validated how HERV transcriptomic analyses can perform that map to larger annotations including more deteriorated HERV sequences.

Our findings are in line with previous studies, showing that indeed cell- and tissue-specific HERV signatures are observable in RNAseq datasets [4, 14, 25, 47, 48]. Here we confirm that differences in HERV transcriptomes between ontologically closely related CD4 and CD8 T cells exist, which can be retrieved from RNAseq datasets with varying technical parameters.

## Conclusion

Locus-specific HERV transcriptomics is a field of research in its beginnings and for which analysis standards yet need to be trialed and established. This study provides to our knowledge the first comprehensive overview of aspects to consider when generating and selecting RNAseq datasets for HERV expression analyses. It provides practical advice concerning technical parameters of suitable datasets and means to combine datasets from studies of different origin. At a time of growing interest in all fields of translational medicine for HERV transcriptomics, our study pinpoints how RNAseq datasets can be explored for cell-type specific HERV transcriptome signatures. We show that while HERV transcriptomic profiles are influenced by study-specific technical aspects both in quality and in quantity, there is considerable overlap of at least 64% in the number of expressed HERVs. Sequencing depth of RNAseq datasets appears to be one critical parameter in view of broad detection of locus-specific HERV transcription. As for CD4 + and CD8 + T cell-specific HERV expression signatures, inter-study differences appear to outweigh biological diversity, making batch effect reduction a necessity when working with multi-sourced datasets. Donor-specific differences can also be compensated for using batch effect corrected input files. In summary, our study provides a first essential guidance of how to select, generate and analyze suitable RNAseq datasets for HERV transcriptomics.

## Electronic supplementary material

Below is the link to the electronic supplementary material.

Supplementary Material 1

Supplementary Material 2

Supplementary Material 3

Supplementary Material 4

## Data Availability

All data is available publically without restrictions under the SRA accession numbers provided in the material and methods section. Project hyperlinks for each study are provided in Table 1.

## Abbreviations

HERV: Human endogenous retrovirus
Mio: Million
Bp: Base pairs
NGS: Next generation sequencing
RNAseq: Ribonucleic acid sequencing
LTR: Long terminal repeat
KZFP: Krüppel-associated box domain-containing zinc-finger protein
SD: Standard deviation
Bc: Batch corrected
PCA: Principal component analysis
HREMP: Human reference epigenome mapping project
