# Supplementary figures and images for: Confounding factors in profiling of locus-specific human endogenous retrovirus (HERV) transcript signatures in primary T cells using multi-study-derived datasets
Source: BMC Med Genomics. 2023 Apr 3;16:68. doi: 10.1186/s12920-023-01486-y (PMC10068191; doi:10.1186/s12920-023-01486-y)

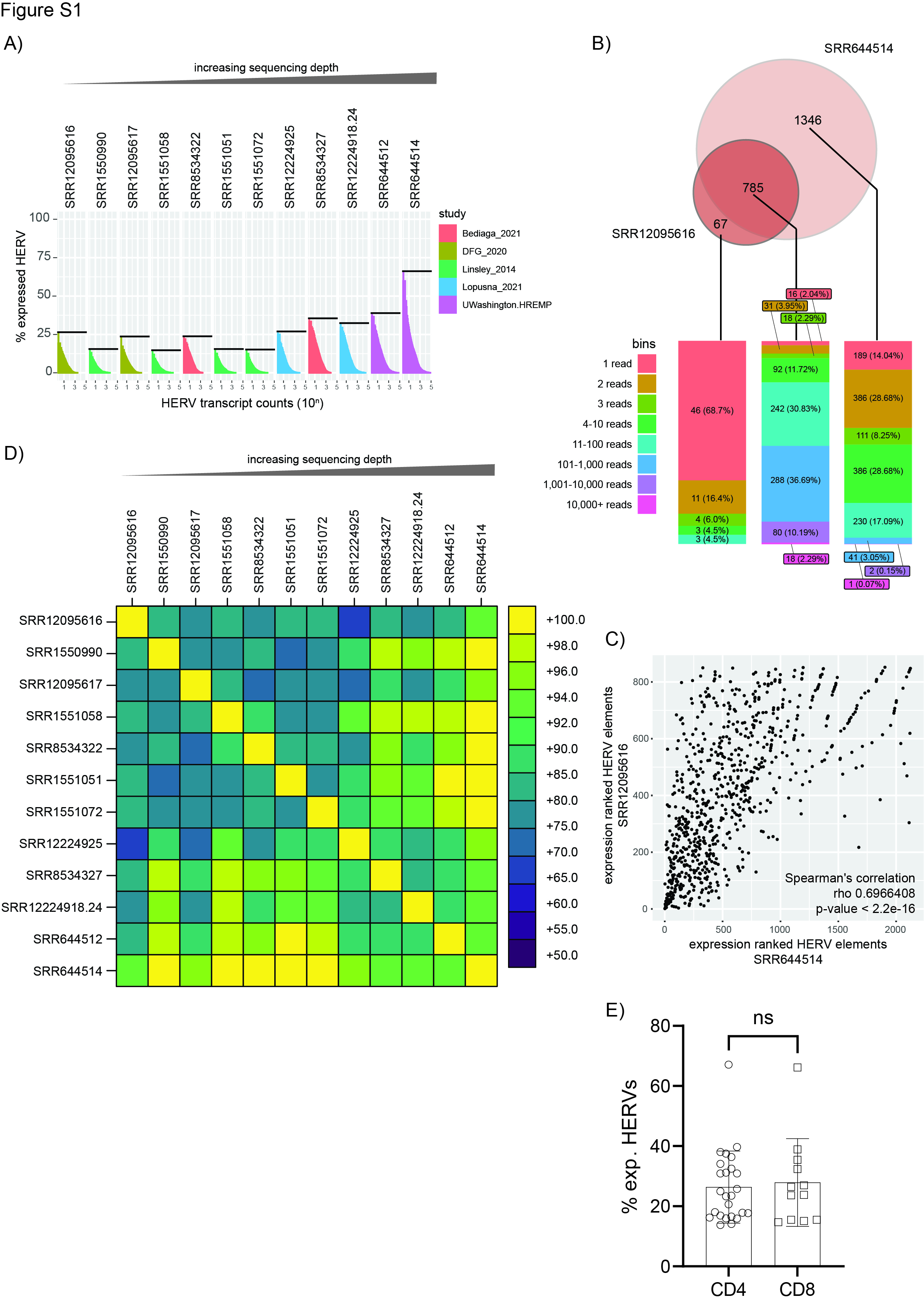

Supplement: Supplementary file 1 — Supplementary Material 1 [file 12920_2023_1486_MOESM1_ESM.jpg]

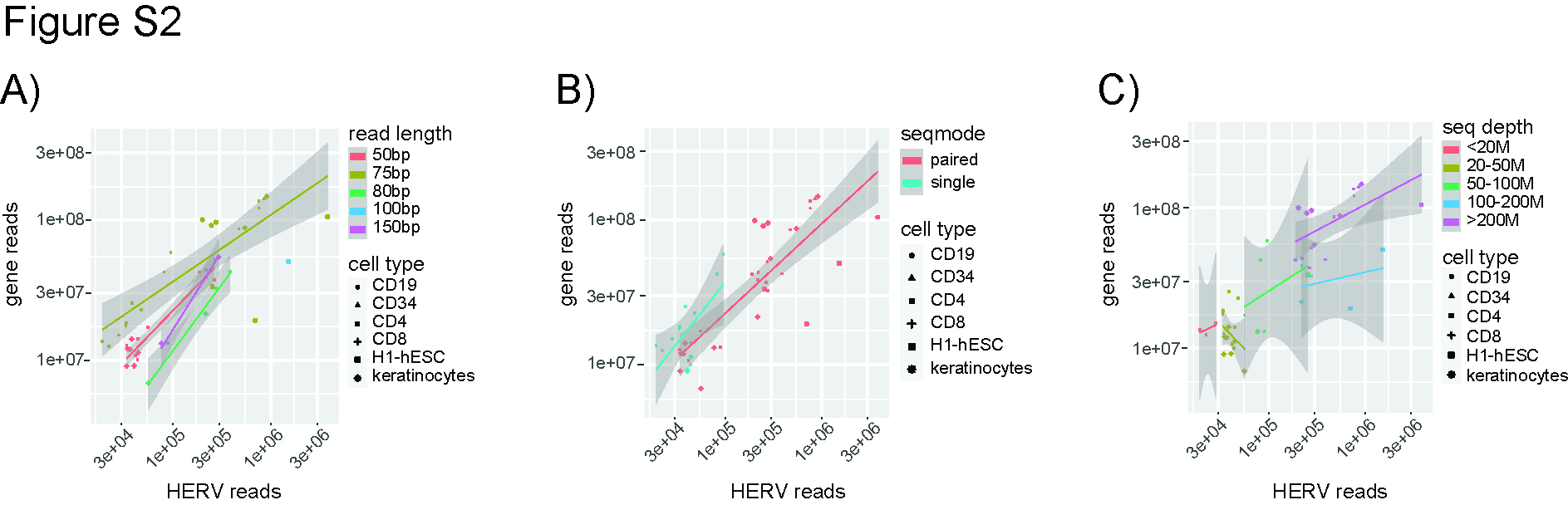

Supplement: Supplementary file 2 — Supplementary Material 2 [file 12920_2023_1486_MOESM2_ESM.jpg]

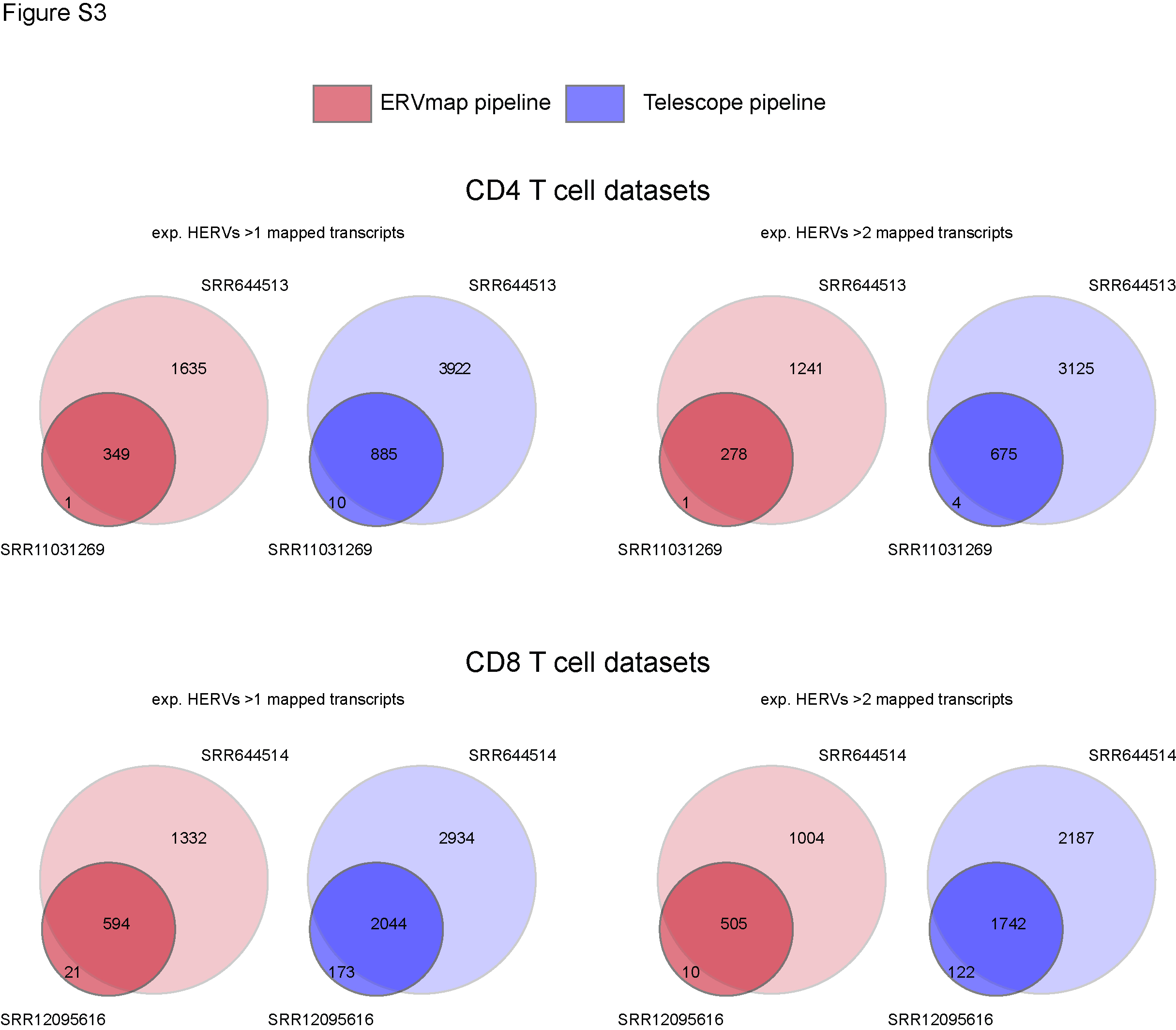

Supplement: Supplementary file 3 — Supplementary Material 3 [file 12920_2023_1486_MOESM3_ESM.jpg]
