# Supplementary material for: Confounding factors in profiling of locus-specific human endogenous retrovirus (HERV) transcript signatures in primary T cells using multi-study-derived datasets
Source: BMC Med Genomics. 2023 Apr 3;16:68. doi: 10.1186/s12920-023-01486-y (PMC10068191; doi:10.1186/s12920-023-01486-y)
Supplement: Supplementary file 4 — Supplementary Material 4 [file 12920_2023_1486_MOESM4_ESM.docx]

**Figure legends for supplementary figures S1, S2 and S3**

*Figure S1: HERV expression in primary CD8+ T cell datasets. A) Raw HERV transcript counts are plotted for each HERV element. A HERV element is considered to be expressed with at least one read being mapped to the HERV loci. Black line indicate % of expressed HERVs per dataset. Datasets are identified by SRA database numbers and ordered by increasing sequencing depth. B) Quantitative comparison of expressed HERV elements between datasets with least and highest sequencing depth. Absolute number of expressed HERV elements are presented in the Venn diagram. Bar chart below depicts distribution of mapped reads per HERV element for each Venn section C) Ranked HERV expression comparison between datasets from B. The Spearman correlation coefficient and p-value is indicated. D) Pairwise comparison matrix presenting the overlap of expressed HERV elements between datasets. Order of datasets equivalent to panel A. E) Relative number of expressed HERV elements in CD4+ and CD8+ T cell datasets. Unpaired two-tailed t-test, ns = not significant.*

*Figure S2: Sequencing parameter impact on HERV transcriptome mapping. Raw mapped HERV and gene transcript counts are plotted and grouped by sequencing parameter read length (A), seqmode (B) and sequencing depth (C).*

*Figure S3: Comparison of analysis pipelines and threshold level on calling expressed HERV elements. Quantitative comparison of expressed HERV elements at various threshold levels between datasets with least and highest sequencing depth for CD4 and CD8 T cell datasets. ERVmap pipeline in red, Telescope pipeline in purple. Number of expressed HERV elements are presented. The ERVmap pipeline includes 3220 near full length HERV elements. Telescope analyses 14,968 HERV elements in total.*
